# Active case-finding for TB among incarcerated women in Peru

**DOI:** 10.5588/ijtld.23.0183

**Published:** 2023-10-01

**Authors:** D. Puma, C. Geadas, R. I. Calderon, C. M. Yuen, J. Jiménez, M. Córdova, B. Martínez, J. Peinado, L. Lecca, M. Tovar

**Affiliations:** 1 Socios En Salud Sucursal Perú, Lima; 2 Facultad de Medicina, Universidad Nacional Mayor de San Marcos, Lima, Perú; 3 Department of Medicine, Brigham and Women’s Hospital, Boston, MA, USA; 4 Grupo de Investigación en Bioquímica y Biología Sintética, Universidad Nacional Federico Villarreal, Lima, Perú; 5 Department of Global Health and Social Medicine, Harvard Medical School, Boston, MA, USA; 6 Instituto Nacional Penitenciario del Perú, Subdirección de Salud Penitenciaria, Lima; 7 Escuela de Medicina, Facultad de Ciencias de la Salud, Universidad Peruana de Ciencias Aplicadas, Lima, Peru

Dear Editor,

The burden of TB is particularly heavy in correctional facilities, driven by overcrowding, a high proportion of people with TB risk factors, and poor sanitation, ventilation and access to medical care.^1^ The prevalence of TB in prisons in South America is estimated to be around 1.7%, and people experiencing incarceration are 27 times more likely than the general population to develop TB.^2^ The WHO recommends using chest radiography (CXR) for routine screening for TB in prisons and penitentiary institutions.^3^ However, many prisons in low- and middle-income countries (LMICs) with high TB burdens lack the infrastructure or resources to do so. Peru has the highest incidence of TB in Latin America,^4^ and routine screening is not systematically performed in prisons: instead, this is driven by the development of symptoms or investigation of an active outbreak. Two recent meta-analyses found that active case-finding efforts in prisons in LMICs detected active TB in 2% of individuals on average,^5^ and that yield varies substantially depending on the screening algorithm used.^6^ Notably, 95% of individuals experiencing incarceration in these studies were male, and none focused specifically on female populations – an important omission. Here we present an analysis of data from an active TB case-finding program using CXR in women’s prisons in Lima, Peru. Our goal was to understand the prevalence of TB to inform potential interventions to reduce its burden in this high-risk population.

We implemented a TB screening program in six women’s prisons in the regions of Lima, La Libertad, Arequipa, and Tacna, from August, 2020 to June, 2021. Free voluntary screening was offered to all women, but they were excluded if they were receiving treatment for active TB. We first asked participants about 1) the presence of symptoms: productive cough lasting longer than 14 days, cough of any other type, weight loss in the preceding 30 days, fever lasting longer than 7 days, and night sweats, 2) personal history of TB in the past, 3) exposure to a person with TB in the preceding 2 years, and 4) history of diabetes mellitus or HIV infection. A digital CXR was obtained for every participant, regardless of symptoms. The findings were analyzed using the artificial intelligence software qXR (qure.AI, Mumbai, India), and classified as normal or abnormal according to the manufacturer’s default settings for TB. Women with abnormal CXR were asked to provide a sputum sample for analysis using Xpert^®^ MTB/RIF Ultra (Ultra; Cepheid, Sunnyvale, CA, USA). Sputum samples were also collected from participants who had normal CXR but had a productive cough lasting longer than 14 days, and participants who had normal CXR with a history of HIV, and had either fever, weight loss, or night sweats. Participants eligible for Ultra testing were also evaluated by a physician for possible clinical diagnosis of TB. Women who were diagnosed with TB were referred to the local health authority for treatment initiation. We calculated the number needed to screen (NNS) to detect one case of TB, defined as the number of radiographs divided by the number of cases of TB diagnosed, and the number needed to test (NNT) to detect one case of microbiologically confirmed TB, defined as the number of Ultra tests divided by the number of positive Ultra results. Written informed consent was obtained from all participants and the study was approved by the institutional review board of the Universidad Peruana Cayetano Heredia, Lima, Peru.

We screened women from six prison facilities and 2,035 met the inclusion criteria and agreed to participate. The median age was 38 years (interquartile range 30–48); 22 women (1.1%) reported living with HIV and 152 (7.5%) reported having diabetes mellitus. A history of prior TB was reported by 127 (6.2%) women, and 386 (19.1%) reported having been exposed to someone with TB in the preceding 2 years. Symptoms were reported by 995 (48.9%) women, productive cough for longer than 14 days by 34 (1.2%), and cough of any type by 154 (15.1%). In addition, 99 (4.9%) had experienced fever for longer than 7 days, 483 (23.9%) had lost weight in the preceding 30 days, and 216 (10.7%) had night sweats. All 2,035 women underwent CXR, of whom 93 (4.6%) met criteria for Ultra testing based on either an abnormal CXR (*n* = 50), normal CXR with productive cough for more than 14 days (*n* = 32), or HIV and fever, weight loss or night sweats (*n* = 7) (Figure). All 93 underwent clinical evaluation, and 88 (94.6%) had Ultra testing. Of these, six (6.8%) had a positive Ultra test and six (6.8%) had a negative test but were diagnosed with TB based on clinical and radiographic evidence. Of the 12 women diagnosed with TB, 2 (16.7%) had self-reported HIV and 2 (16.7%) had self-reported diabetes. All 12 initiated anti-TB therapy, including two with rifampin-resistant TB. The NNS was 170, and the NNT was 15.

**Figure:**
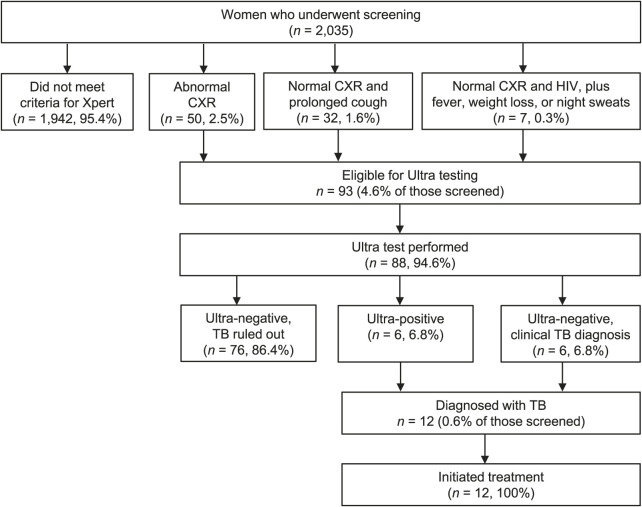
Screening and diagnosis of active TB disease in 2,035 women in six prisons in Peru. CXR = chest radiography; Ultra = Xpert^®^ MTB/RIF Ultra (Cepheid, Sunnyvale, CA, USA).

During an active case-finding program, we diagnosed TB in 0.6% of the women screened in prisons in Peru. To the best of our knowledge, this is the first report of active case-finding focused on TB in incarcerated women. Although the yield of TB is lower than that reported for men in prisons in Peru,^7^ it is substantially higher than TB incidence in the general population (130 per 100,000).^4^ These findings highlight the importance of routine and systematic screening for this particularly vulnerable population using comprehensive algorithms that investigate for clinical, radiographic, and microbiologic evidence of TB.

There are limitations to using our screening program data to draw conclusions about TB prevalence among women in prison in general. First, the intervention took place during the COVID-19 pandemic and to reduce crowding and protect those at highest risk, the government allowed for early release of individuals with comorbidities, who would have also been at higher risk of TB.^8^ Second, we did not perform Ultra testing for everyone, nor did we have the resources to perform mycobacterial culture. Finally, we did not assess the number of women already on TB treatment in the prison, who were excluded from our screening intervention. Together, these limitations suggest that the prevalence of TB detected among our screened population is likely an underestimate.

We found a significant burden of TB among females in prison in Peru. Routine and systematic strategies of active case-finding using CXR, followed by molecular testing at entry and periodically during incarceration may increase timely TB detection. This could help reduce transmission, morbidity, and mortality in this high-risk vulnerable population.

## References

[1] Fazel S, Baillargeon J. (2011). The health of prisoners. Lancet.

[2] Cords O (2021). Incidence and prevalence of tuberculosis in incarcerated populations: a systematic review and meta-analysis. Lancet Public Health.

[3] World Health Organization (2021). WHO operational handbook on tuberculosis Module 2: Screening – Systematic screening for tuberculosis disease.

[4] World Health Organization (2022). Global tuberculosis report, 2022.

[5] Bohlbro AS (2021). Active case-finding of tuberculosis in general populations and at-risk groups: a systematic review and meta-analysis. Eur Respir J.

[6] Naufal F (2022). Number needed to screen for TB in clinical, structural or occupational risk groups. Int J Tuberc Lung Dis.

[7] Salazar-De La Cuba AL (2018). High prevalence of self-reported tuberculosis and associated factors in a nation-wide census among prison inmates in Peru. Trop Med Int Health.

[8] Government of Peru (2020). Decreto Supremo que establece supuestos especiales para la evaluación y propuesta de recomendación de Gracias Presidenciales, y determina su procedimiento, en el marco de la emergencia sanitaria por COVID-19. https://busquedas.elperuano.pe/normaslegales/decreto-supremo-que-establece-supuestos-especiales-para-la-e-decreto-supremo-n-004-2020-jus-1865717-3/.

